# Malnutrition and Nutrition Impact Symptoms in Kuwaiti Colorectal Cancer Patients: Validation of PG-SGA Short Form

**DOI:** 10.3390/nu17172770

**Published:** 2025-08-27

**Authors:** Raghad Obaid, Dalal Alkazemi

**Affiliations:** 1 Kuwait Cancer Control Center, P.O. Box 42263, Shuwaikh 70654, Kuwait; raghad.obaid@grad.ku.edu.kw; 2 Department of Food Science and Nutrition, College of Life Sciences, Kuwait University, Shadadiya, P.O. Box 5969, Safat 13060, Kuwait

**Keywords:** malnutrition, colorectal cancer, PG-SGA, nutrition impact symptoms, Kuwait, nutritional screening, PG-SGA SF, MST

## Abstract

**Background/Objectives:** Malnutrition is a common but underrecognized complication in colorectal cancer (CRC), contributing to poor treatment outcomes and reduced quality of life. Regional data from the Gulf remains limited. This study assessed the prevalence of malnutrition and nutrition impact symptoms (NISs) among CRC patients in Kuwait. It evaluated the diagnostic performance of the PG-SGA Short Form (PG-SGA SF) in comparison to the full PG-SGA and the Malnutrition Screening Tool (MST). **Methods:** A cross-sectional study was conducted among 65 CRC outpatients at the Kuwait Cancer Control Center. Nutritional status was assessed using the full PG-SGA, PG-SGA SF, and MST. Dietary intake, anthropometry, biochemical parameters, and NISs were collected. Logistic regression identified independent predictors of malnutrition, and the performance of the tool was evaluated using kappa statistics and diagnostic accuracy metrics. **Results:** Malnutrition (PG-SGA B/C) was identified in 61.4% of patients. Loss of appetite, dry mouth, and nausea were significantly associated with malnutrition (*p* < 0.00385); dry mouth independently predicted malnutrition (OR: 17.65, 95% CI: 2.02–154.19, *p* = 0.009). BMI was not predictive, but reduced mid-arm circumference was significantly associated. PG-SGA SF showed strong agreement with the full PG-SGA (κ = 0.75), with high sensitivity (87.2%) and specificity (88.5%), outperforming MST (κ = 0.38). Only 23.5% of moderately malnourished patients were referred to a dietitian. **Conclusions:** Malnutrition and NIS are highly prevalent among Kuwaiti CRC patients. PG-SGA SF is a valid and efficient screening tool that should replace MST in oncology settings. Symptom-informed screening and structured referral protocols are crucial for enhancing nutrition care.

## 1. Introduction

Cancer remains a leading cause of morbidity and mortality worldwide, with colorectal cancer (CRC) ranking among the most prevalent malignancies globally [1]. In Kuwait, cancer is the second most common cause of death after cardiovascular disease, with CRC representing the second most diagnosed cancer in males after prostate cancer and the third in females after breast and thyroid cancers. It is also the second leading cause of cancer-related mortality in both genders [2,3].

Malnutrition is a frequent complication in CRC patients. It is associated with adverse clinical outcomes, including reduced quality of life, impaired treatment response, prolonged hospital stays, higher rates of re-admission, and increased healthcare costs [4,5,6]. Its prevalence among oncology patients ranges from 20% to over 70%, depending on cancer type, disease stage, and the nutritional assessment tool employed [7].

CRC patients are particularly vulnerable to malnutrition due to disease-related symptoms and treatment side effects. Nutrition impact symptoms (NISs)—such as anorexia, nausea, vomiting, dry mouth, mouth sores, early satiety, fatigue, and swallowing difficulties—directly impair oral intake and contribute to progressive nutritional decline [8]. Managing these symptoms effectively is critical for preventing deterioration and optimizing clinical outcomes [9].

The Patient-Generated Subjective Global Assessment (PG-SGA) is the gold standard for assessing nutritional status in oncology settings. It enables the detection of varying degrees of malnutrition and guides intervention strategies [10]. Screening tools, such as the Malnutrition Screening Tool (MST), also support the early identification of patients at risk, facilitating timely referrals and nutritional care [4]. A validated abbreviated version, known as the PG-SGA Short Form (PG-SGA SF), includes only the patient-reported components—weight changes, food intake, symptom burden, and functional status—and is increasingly recommended for use in busy clinical settings as a rapid nutritional screening tool [11]. Despite international recommendations for early nutritional assessment in oncology, regional data on malnutrition and its symptomatology among CRC patients in Kuwait remain limited. Most Middle Eastern studies have focused on cancer patients in general without distinguishing between cancer types or examining specific symptoms affecting dietary intake. No published research has investigated the nutritional status or prevalence of nutrition impact symptoms (NISs) specifically in CRC patients within Kuwait.

This study addresses this gap by evaluating the nutritional status of CRC patients at the Kuwait Cancer Control Center (KCCC), identifying NISs significantly associated with malnutrition, and assessing the agreement between the MST and PG-SGA tools. The findings underscore the importance of incorporating comprehensive nutritional assessment protocols into routine oncology care to enhance patient outcomes and quality of life.

## 2. Materials and Methods

### 2.1. Study Design and Inclusion Criteria

A cross-sectional study was conducted at KCCC from January 2024 to July 2024. Eligible participants included colorectal cancer patients aged 18 years or older of both genders who were attending the outpatient clinic for physician follow-up and actively receiving treatment. Patients were excluded if they had end-stage cancer with less than three months of expected survival, mental disabilities impairing comprehension, an inability to stand for anthropometric measurements, failure to communicate effectively in either Arabic or English, or refusal to participate.

### 2.2. Data Collection

Data were collected collaboratively by a trained clinical dietitian and a medical oncologist from the Gastrointestinal Oncology Department at the KCCC between January and July 2024. The data collection process occurred in two phases, beginning in the outpatient clinic and continuing in the chemotherapy daycare unit. In the outpatient setting, patients were first screened for malnutrition risk using the MST. Anthropometric measurements were taken, including weight, height, mid-arm circumference (MAC), and mid-calf circumference (MCC). Mid-arm and mid-calf circumference methods were selected due to their feasibility, low cost, and minimal equipment requirements in a busy outpatient oncology setting, particularly where advanced body composition assessments such as bioelectrical impedance analysis or CT scans are not routinely available. The medical oncologist then performed a nutrition-focused physical examination (NFPE) to support the comprehensive nutritional assessment required for the full PG-SGA evaluation. The second phase took place at the chemotherapy daycare unit, where the clinical dietitian conducted structured, face-to-face interviews with each participant using a detailed survey developed explicitly for this study. The survey captured demographic characteristics, medical and treatment history, dietary patterns, symptom burden, and nutritional care practices. Additional clinical information, including biochemical parameters and treatment details, was extracted from each patient’s electronic medical records through the Hospital Information System (HIS).

### 2.3. Survey Tool and Data Components

The survey tool collected demographic data (gender, age, nationality, marital status, income, and education), medical history (chronic conditions, surgeries, cancer type and stage, diagnosis date, treatment regimens, stoma status, and medication and supplement use), and nutritional history (feeding type, diet consistency, oral nutritional supplements, nutrition impact symptoms, food intolerances, MST scores, prior dietitian referrals, and PG-SGA classifications). Anthropometric measurements included current and usual body weight; height; mid-arm and mid-calf circumference; BMI; and weight changes over the past one, three, and six months. Relevant biochemical data encompassed liver and renal function, electrolyte levels, and complete blood count.

### 2.4. Dietary Intake Method

Dietary intake was assessed using three non-consecutive 24 h dietary recalls—two on weekdays and one on a weekend—administered by trained dietitians using the multi-pass method [12]. Participants reported all foods, beverages, and snacks consumed in the previous 24 h, including portion sizes, preparation methods, and brand names when applicable. Household measures and visual aids were used to enhance accuracy. While this method is widely accepted for estimating usual intake, its validity in oncology populations is challenged by day-to-day intake variability due to fluctuating appetite, nausea, early satiety, and other treatment-related symptoms. Despite using structured interviews and visual prompts, recall bias and misreporting of intake remain possible. Prior studies suggest that incorporating food diaries or objective biomarkers alongside recalls may improve the reliability of dietary assessments in cancer patients, particularly when nutrition impact symptoms are present [12,13,14,15].

### 2.5. Assessment of Nutritional Status

The risk of malnutrition was initially assessed using the Malnutrition Screening Tool (MST), a validated two-item instrument focused on recent unintentional weight loss and decreased appetite. A score of 2 or greater indicated nutritional risk. Comprehensive nutritional status was subsequently evaluated using the Patient-Generated Subjective Global Assessment (PG-SGA), widely regarded as the gold standard in oncology nutrition. For clarity, this tool is referred to as the full PG-SGA throughout the manuscript. The full PG-SGA assesses recent weight changes, dietary intake, nutrition impact symptoms (NISs), and functional status and includes a physical examination to detect muscle wasting, fat loss, or fluid retention. A medical oncologist performed a nutrition-focused physical examination (NFPE), and PG-SGA scores were calculated using a validated online platform, categorizing patients as well nourished (PG-SGA A), moderately malnourished (PG-SGA B), or severely malnourished (PG-SGA C) [7,16]. For analysis, PG-SGA categories B and C were grouped as malnourished.

In addition to the full PG-SGA, data were used to retrospectively calculate the PG-SGA Short Form (PG-SGA SF), which comprises the patient-reported components (Boxes 1–4): weight history, food intake, symptom burden, and functional capacity. In contrast to the MST, the PG-SGA SF includes additional domains such as nutrition impact symptoms, functional capacity, and patient self-assessment. A detailed comparison of the two tools is provided in Supplementary Table S1. The PG-SGA SF is increasingly recommended for routine use in oncology due to its brevity and strong diagnostic performance [16]. Following previous validation studies, a PG-SGA SF score of ≥9 was used to define high nutritional risk and identify patients requiring dietitian referral [17,18]. This threshold was also applied in the multivariate regression analysis and underpins the clinical referral protocol proposed in the discussion. This allowed for a comparative analysis of the screening performance of the PG-SGA SF and MST against the full PG-SGA.

### 2.6. Dietary Assessment

Dietary data were analyzed using the Nutritics software (version 6.02, Dublin, Ireland). Energy requirements were estimated at 25 kcal/kg/day, a widely accepted standard for oncology patients undergoing treatment [7]. Actual caloric intake was compared against these calculated requirements to assess adequacy. For underweight patients (BMI < 18.5 kg/m^2^), ideal body weight (IBW) was used. In comparison, for obese patients (BMI ≥ 30 kg/m^2^), adjusted body weight (ABW) was applied to improve accuracy in estimating metabolic needs [19]. IBW and ABW calculations were performed using the MDCalc mobile application (version 5.4.0; MD Aware LLC), a practical tool routinely used in clinical dietetics at KCCC. Protein requirements were set at ≥1.2 g/kg body weight per day, reflecting guidelines for cancer patients to preserve lean mass and support metabolic demands [7,20]. As with energy calculations, IBW and ABW were used as appropriate. Patients were classified as meeting requirements if their intake reached ≥25 kcal/kg and ≥1.2 g/kg of protein.

### 2.7. Missing Data Handling

Incomplete data were handled using case-wise exclusion for specific analyses. Patients with missing dietary recall entries (e.g., fewer than two complete 24 h recalls) or incomplete laboratory values were excluded only from the respective analyses to preserve statistical power in other sections. The final sample size for each analysis is reported where applicable.

### 2.8. Statistical Analysis

Data were analyzed using the R statistical software (version 4.4.3). Descriptive statistics were used to summarize patient demographics, clinical characteristics, and nutritional status. Categorical variables were reported as frequencies and percentages. Associations between categorical variables, such as NISs and malnutrition status, were assessed using the chi-square test, with statistical significance set at *p* < 0.05. To control for multiple comparisons across 13 NIS variables, Bonferroni correction was applied, adjusting the significance threshold to α = 0.00385. Only *p*-values below this corrected threshold were considered statistically significant. Non-significant but clinically notable trends (e.g., early satiety, *p* = 0.0116) were described as relevant observations. Differences in continuous variables, such as duration since diagnosis across cancer stages (early locally advanced, late locally advanced, and metastasized), were examined using one-way ANOVA (Welch’s test) to account for heteroscedasticity.

## 3. Results

### 3.1. Recruitment and Sample Size Considerations

**Figure 1:**
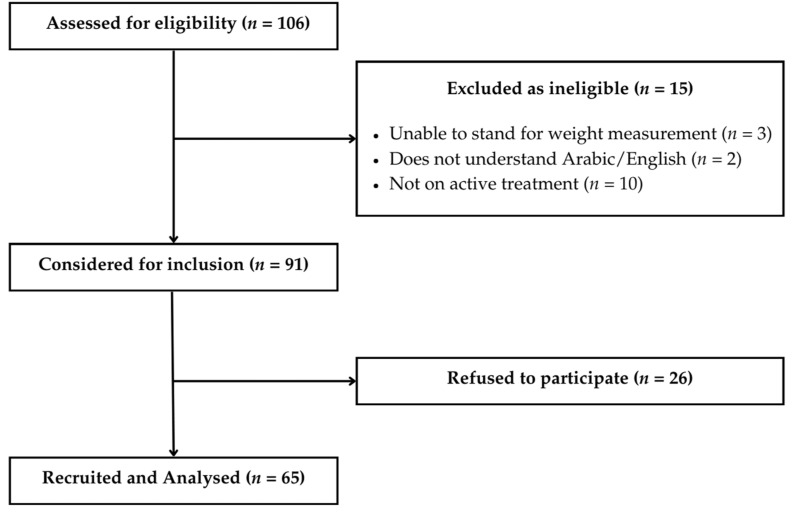
Participant flow chart.

Of the 106 CRC patients initially screened, 91 met the eligibility criteria. After obtaining informed consent, 65 patients were enrolled and completed all assessments. The final sample size (*n* = 65) aligns with similar observational studies in oncology nutrition that utilized cross-sectional designs to assess the prevalence of malnutrition and its associations with symptoms [15,21]. However, no formal a priori power calculation was performed, and the modest sample size may have limited the ability to detect significant differences in subgroup analyses or perform stratified analyses (e.g., by stage, gender, or treatment modality). This should be considered when interpreting the generalizability and strength of associations. The participant flow is depicted in Figure 1.

### 3.2. Sociodemographic Characteristics

The sociodemographic profiles of the participants are detailed in Table 1. The sample consisted of 65 patients with colorectal cancer, with a slightly higher proportion of males (56.9%) than females (43.1%). Most participants were married (86.2%) and ranged in age from 40 to 74 years. The majority resided in urban governorates, were Kuwaiti nationals (49.2%), and held at least a high school education (56.9%). The income distribution showed that over half (53.8%) reported a monthly income exceeding KWD 1000. No statistically significant associations were found between nutritional status (based on PG-SGA classification) and any sociodemographic variables, including gender (*p* = 0.89), marital status (*p* = 0.12), age (*p* = 0.08), governorate (*p* = 0.15), employment status (*p* = 0.42), income (*p* = 0.68), nationality (*p* = 1.00), or educational level (*p* = 0.28).

### 3.3. Clinical Characteristics by Nutritional Status

Table 2 summarizes the clinical characteristics of the colorectal cancer (CRC) patients by nutritional status, as classified by the full PG-SGA. While no statistically significant differences were observed across most variables, several notable trends emerged. Patients with two or more comorbidities, those with a stoma, and current smokers were more frequently classified as malnourished, although these associations did not reach statistical significance. The presence of a stoma showed a borderline association with malnutrition (*p* = 0.050). Regarding BMI, malnutrition was observed even among patients in the overweight and obese categories, with 38.9% and 55.0%, respectively, classified as malnourished, highlighting the limitations of BMI in capturing nutritional risk. Cancer stage and treatment type were not significantly associated with nutritional status.

### 3.4. Nutritional Status of CRC Patients

According to the full PG-SGA, 60% of patients were classified as malnourished (Table 3), with 24.6% classified as moderately malnourished and 35.4% as severely malnourished. The PG-SGA SF identified 56.9% of patients as being at risk (Table 3), whereas the MST identified 64.6% of patients as being at nutritional risk (Table 4). Malnourished patients had substantially higher symptom burden and lower functional capacity scores compared with well-nourished patients. After Bonferroni correction, loss of appetite, dry mouth, and nausea remained significantly associated with malnutrition. Detailed median scores for individual PG-SGA SF components and full NIS frequencies are provided in Supplementary Table S2. These findings support the use of PG-SGA SF as a practical tool for identifying nutritional risk in CRC outpatients.

### 3.5. Anthropometrics and Malnutrition

As shown in Table 5, several anthropometric measures were significantly associated with malnutrition status. Malnourished patients experienced a greater percentage weight loss in both the past month (median 1.25% vs. 0.00%, *p* = 0.001) and 6 months (3.33% vs. 0.00%, *p* = 0.003) compared to well-nourished individuals. Mid-arm circumference was significantly lower among the malnourished (27.35 cm vs. 31.20 cm, *p* = 0.01), as was mid-calf circumference (34.40 cm vs. 37.72 cm, *p* = 0.04). Mid-arm circumference was selected as a primary anthropometric measure due to its simplicity, low cost, and practicality in busy oncology clinics, where advanced body composition techniques such as CT and bioelectrical impedance are not routinely accessible. While less precise than CT bioelectrical impedance, MAMC is validated in oncology populations and demonstrates a strong correlation with muscle mass, supporting its use in routine clinical practice. The current body weight also differed significantly between groups (*p* = 0.049). In contrast, no significant differences were observed in BMI (*p* = 0.15) or absolute weight at 1, 3, or 6 months prior, suggesting that percentage weight loss and circumferential measures may be more sensitive indicators of malnutrition in this population than BMI or body weight alone.

### 3.6. Dietary Intake and Malnutrition

As shown in Table 6, nutrient intake differed significantly between malnourished and well-nourished patients. Energy and protein intakes were significantly lower among malnourished patients, both in absolute terms and relative to body weight (Table 6). The proportion meeting estimated energy requirements was low in both groups and did not differ significantly. Some patients with inadequate intake were not classified as malnourished because the PG-SGA incorporates multiple domains beyond intake, including recent weight change, physical function, and symptom burden. A post hoc power analysis for this comparison yielded an effect size (w) of 0.41 (moderate) and an achieved power of 79.9%, indicating the study was marginally underpowered in detecting a statistically significant association at the conventional 80% threshold. While the *p*-value is not statistically significant, a moderate effect size suggests potential clinical relevance, warranting further investigation in larger samples. The complete dataset, including energy adequacy proportions, is presented in Supplementary Table S3, and the detailed macronutrient distributions and non-significant comparisons are provided in Supplementary Table S4.

### 3.7. Biochemical Data

Several biochemical markers were significantly associated with malnutrition status (Supplementary Table S5). Malnourished patients exhibited higher neutrophil percentages (63.9% vs. 52.3%, *p* = 0.004) and lower lymphocyte percentages (23.1% vs. 31.3%, *p* = 0.02), suggesting possible immune dysregulation. Serum magnesium was also significantly lower in malnourished individuals (0.76 mmol/L vs. 0.84 mmol/L, *p* = 0.02). Additionally, red blood cell count (RBC) was reduced in the malnourished group (4.15 vs. 4.62 × 10^12^/L, *p* = 0.05), potentially reflecting early hematologic effects of malnutrition. Other laboratory markers, including hemoglobin, albumin, total protein, and inflammatory enzymes, showed trends toward lower values in malnourished patients but did not reach statistical significance (*p* > 0.05). These findings indicate that while some immune and electrolyte-related parameters may reflect nutritional deterioration, many routine biochemical values may lack sensitivity in detecting malnutrition in oncology outpatients.

### 3.8. Referral to a Dietitian and Malnutrition Status

Dietitian referrals increased with the severity of malnutrition, as shown in Table 7: 65.2% of patients with severe malnutrition were referred, compared to 23.5% with moderate malnutrition and 32% of well-nourished patients (*p* = 0.001). However, when malnourished (moderate and severe) patients were grouped and compared to well-nourished patients, the overall difference in referral rates was not statistically significant (70.4% vs. 52.6%, *p* = 0.24). Despite this, post hoc power analysis indicated a large effect size (w = 1.44) and 100% power, suggesting the nonsignificant result may be due to sample imbalance or clinical heterogeneity rather than lack of association.

### 3.9. NIS According to Malnutrition Status

As shown in Table 8, several NISs were significantly more prevalent among malnourished patients as classified by the PG-SGA. The most common symptoms were loss of appetite (69.2% vs. 23.1%, *p* < 0.001), followed by dry mouth (48.7% vs. 3.8%, *p* < 0.001) and nausea (35.9% vs. 0%, *p* < 0.01). Other symptoms with notable differences included early satiety (33.3% vs. 3.8%, *p* = 0.012), diarrhea (41.0% vs. 11.5%, *p* = 0.023), and mouth sores (23.1% vs. 0%, *p* = 0.023). Vomiting and fatigue occurred exclusively in malnourished patients. After applying Bonferroni correction (*p* < 0.00385), only loss of appetite, dry mouth, and nausea remained statistically significant. These findings underscore the strong association between specific NIS and malnutrition, highlighting their clinical value in identifying patients at risk for CRC.

### 3.10. Predictors of Malnutrition

Multivariate logistic regression was used to examine factors associated with malnutrition (PG-SGA B/C vs. A). As shown in Table 9, dry mouth was a strong independent predictor, with patients reporting this symptom having significantly higher odds of being malnourished (OR: 17.65; 95% CI: 2.02–154.19; *p* = 0.009). Low mid-arm muscle circumference was also associated with increased risk (OR: 5.21; 95% CI: 1.25–21.78; *p* = 0.023). Other covariates, including BMI, stoma presence, cancer stage, sex, and age, were not statistically significant. Symptoms such as vomiting and fatigue were excluded due to perfect separation, as they were present only among malnourished patients, preventing model convergence. While vomiting and fatigue were excluded from the regression model due to perfect separation, their exclusive presence among malnourished patients suggests they may be strong clinical indicators. Exclusion from the model likely underestimates their predictive value, and future studies with larger samples could better quantify their contribution. The final model demonstrated strong performance, with an area under the ROC curve (AUC) of 0.842, suggesting good discriminative ability. The model’s Nagelkerke R^2^ was 0.398, indicating that approximately 40% of the variance in malnutrition status was explained by the included predictors. The Akaike Information Criterion (AIC) for the model was 61.2, indicating a reasonable model fit relative to its complexity.

### 3.11. Agreement Between Screening and Assessment Tools

Compared to the full PG-SGA, the PG-SGA SF demonstrated superior diagnostic accuracy, with high sensitivity, specificity, and predictive values (Table 10). Agreement was substantial (κ = 0.75; 95% CI: 0.58–0.91), reflecting strong consistency in classifying nutritional risk. In contrast, the MST showed worse performance and only fair agreement with the full PG-SGA (κ = 0.38; 95% CI: 0.14–0.61), indicating greater variability and reduced reliability.

## 4. Discussion

This study reveals a critically high prevalence of malnutrition (61.4%) among CRC patients at the KCCC, as determined by the PG-SGA, the established gold standard for nutritional assessment in oncology [10]. This finding aligns with global reports of malnutrition rates ranging from 20% to 70% [7,22,23] and is further supported by recent multicenter data reporting similarly high rates in gastrointestinal cancer populations [24,25]. Notably, the malnutrition rate observed in Kuwait exceeds the 51.8% reported among chemotherapy patients in Riyadh [26], suggesting potential additional or unaddressed nutritional vulnerabilities in the Kuwaiti population. Given the established consequences of malnutrition, including impaired immunity, reduced treatment tolerance, prolonged hospital stays, and diminished quality of life [7], these findings underscore the urgent need for systematic nutritional screening and early intervention within oncology care pathways in Kuwait and the Gulf region.

A key finding was the strong association between NIS and the severity of malnutrition. Patients with severe malnutrition reported a significantly higher symptom burden, particularly anorexia, dry mouth, and nausea, consistent with prior research linking symptom load to nutritional decline [8,10,27,28]. Following Bonferroni correction (applied to control for multiple NIS comparisons), only loss of appetite, dry mouth, and nausea remained significantly associated, highlighting their potential as key indicators of early nutritional risk. In multivariate analysis, dry mouth emerged as a statistically significant independent predictor, while anorexia approached significance. Notably, vomiting and fatigue were reported exclusively by malnourished patients, reinforcing their clinical relevance. These results support the integration of routine NIS screening into nutritional assessments for early identification of at-risk patients.

Anthropometric measures provided further evidence of nutritional compromise. Significant reductions in mid-arm and mid-calf circumferences, along with greater six-month weight loss, correlated with increasing malnutrition severity. Crucially, BMI did not differ significantly across nutritional status groups, with many malnourished patients falling within normal, overweight, or obese categories. This highlights a critical limitation of BMI in oncology: its inability to account for changes in body composition, particularly the depletion of muscle mass. Evidence suggests that nutritional decline often precedes visible signs, such as weight loss, especially in patients with CRC, where sarcopenic obesity (concurrent excess adiposity and skeletal muscle loss) can mask severe muscle wasting and functional decline [21,29,30]. These results emphasize the necessity of multidimensional tools, such as the PG-SGA, which capture weight change, muscle wasting, symptom burden, and functional status beyond BMI alone [10,31]. In practice, incorporating simple, low-cost measures like mid-arm and mid-calf circumferences into routine outpatient assessments can help identify muscle loss early, even when BMI appears normal.

In multivariate regression, dry mouth was the strongest independent predictor of malnutrition, with an adjusted OR of 17.65 (95% CI: 2.02–154.19, *p* = 0.009), supporting the existing literature that links oral symptoms to nutritional decline in cancer patients [8,27]. Likewise, reduced MAMC was independently associated with an increased risk of malnutrition, aligning with prior findings that muscle loss predicts poor outcomes and decreased survival in metastatic colorectal cancer [21]. In contrast, BMI was not a significant predictor, reaffirming its limited utility as a standalone marker in oncology, especially in patients with sarcopenic obesity [30].

Vomiting and fatigue demonstrated perfect separation in the dataset, occurring exclusively in malnourished patients. Although excluded from the final regression model due to statistical constraints, their presence further supports symptom burden as a meaningful clinical marker of nutritional risk. The regression model demonstrated strong discriminative performance (AUC = 0.842, Nagelkerke R^2^ = 0.398, and AIC = 61.2), reinforcing the value of incorporating both NIS and anthropometric measures into routine nutritional assessments, in line with international guidelines recommending comprehensive, symptom-informed screening [7].

Our data revealed that despite evidence favoring early nutritional care, referrals to dietitians were disproportionately made for severely malnourished patients, while only 23.5% of those with moderate malnutrition were referred. Notably, even among those classified as severely malnourished, a proportion were not receiving any form of nutritional support at the time of assessment, suggesting both delayed and missed interventions. Findings from clinical practice indicate that, despite growing awareness, malnutrition remains inadequately addressed in oncology care settings [32], with oncologists often overlooking early signs or delaying referrals until advanced deterioration [33]. This reactive pattern, delaying intervention until advanced deterioration, is concerning given evidence that early, structured nutritional support improves outcomes [8]. Late referrals based on visual cues rather than validated tools are compounded by limited dietetic staffing, inconsistent protocols allowing moderate cases to be missed, insufficient clinician training in recognizing functional malnutrition (particularly in patients with a normal or high BMI), and a lack of understanding of the significance of early NISs [34]. Initiating support only after severe signs appear reduces the effectiveness of therapy and negatively impacts treatment outcomes, leading to increased morbidity, prolonged hospital stays, and diminished quality of life [35,36].

Although the association between meeting energy requirements and PG-SGA status was not statistically significant, the higher prevalence of severe malnutrition among those with insufficient intake suggests clinical relevance. This aligns with findings linking low energy intake (<25 kcal/kg/day) to muscle depletion in gastric and CRC patients [37], reinforcing the idea that inadequate intake contributes directly to muscle wasting and adverse outcomes, further supporting the need for tools like the PG-SGA that assess intake. Integrating food diaries alongside dietary recalls could enhance the accuracy of intake assessment in future research, particularly in patients with fluctuating symptoms.

When comparing screening tools, the PG-SGA SF demonstrated superior diagnostic accuracy and agreement with the full PG-SGA compared to the MST. The PG-SGA SF outperformed the MST in diagnostic accuracy and agreement, reducing false positives and improving early identification of at-risk patients. While the MST is quick, its limited precision risks unnecessary referrals, straining resources, and potentially delaying care for truly malnourished patients in resource-constrained settings. Our findings support prior validations of the PG-SGA SF in oncology [18] and call into question the reliability of the MST as a standalone oncology screener [4,10]. We recommend replacing the MST with the PG-SGA SF in Kuwaiti CRC clinics due to its accuracy, feasibility, and patient acceptability. The MST could serve as preliminary triage only where PG-SGA SF resources are unavailable, with positive results confirmed by the PG-SGA SF. While our findings support the routine use of the PG-SGA SF, formal cost-effectiveness evaluations in the Kuwaiti context are needed to determine the potential savings associated with reducing false positives and enabling earlier nutritional interventions.

These findings highlight an urgent need for structured, proactive nutrition care pathways in Kuwaiti oncology practice. Implementing the following evidence-based strategies is crucial: (1) Mandatory PG-SGA SF screening at the first oncology visit, leveraging its high diagnostic accuracy (κ = 0.747) and patient-administered format, minimizing staff burden; (2) EHR-embedded referral triggers for PG-SGA SF scores ≥ 9 to automate dietitian consultations, reducing reliance on clinician discretion; (3) integration of dietitians into multidisciplinary tumor boards for early, coordinated care; (4) staff training focusing on BMI limitations, recognizing sarcopenia/functional indicators (e.g., reduced grip strength) and early NISs; (5) standardized referral protocols based on objective screening results. Regional adoption of such protocols, mirroring the success in Jordanian and Palestinian centers (e.g., a 30% reduction in malnutrition via algorithm-driven referrals [26]), is recommended. While full EHR automation may require upgrades, initiating standardized workflows at the KCCC is a feasible option [38]. Although the PG-SGA SF is brief and patient-completed, implementation in non-digital or resource-limited settings may require practical adaptations. Paper-based PG-SGA SF forms with color-coded risk categories can facilitate consistent screening, while low-cost offline scoring apps offer a feasible alternative in clinics without EHR integration. Centralizing screening responsibility with trained nurses can ease the burden on oncologists and help ensure timely referrals and intervention.

Study limitations include the single-center design and modest sample size, which may limit the generalizability of the findings. Recruitment from outpatient clinics may underestimate the prevalence by excluding inpatients with greater complexity or severity. Incomplete dietary recalls and missing biochemical parameters reduced sample sizes for some analyses. The conservative Bonferroni correction may have increased Type II error risk for NIS associations. Despite these limitations, this is the first study in Kuwait documenting high malnutrition prevalence and NIS burden among CRC outpatients, providing valuable insights.

## 5. Conclusions

This study highlights the high burden of malnutrition and NISs among Kuwaiti CRC patients. It validates the PG-SGA SF as a robust screening tool and underscores the clinical utility of symptom-based screening (particularly anorexia, dry mouth, and nausea) for early risk detection. Critically, it identifies a significant gap in proactive care, evidenced by inadequate referrals for moderate malnutrition due to systemic barriers. Addressing this through structured pathways—incorporating mandatory PG-SGA SF screening, automated EHR referrals, dietitian integration, staff training, and standardized protocols—is urgently needed to enable early intervention, improve treatment tolerance, reduce complications, and enhance patient outcomes. While Kuwaiti hospitals, such as the KCCC, have EHR infrastructure, they lack embedded nutrition modules or automated triggers; modest enhancements could facilitate PG-SGA SF-based alerts. Future research should validate these findings in larger, multicenter Kuwaiti and Gulf cohorts and evaluate the impact of implementing systematic nutrition support within comprehensive cancer care.

## Figures and Tables

**Table 1:** Sociodemographic characteristics of colorectal cancer patients—total sample, *n* = 65—according to nutritional status based on full PG-SGA.

		N (%)	Malnourished	Well Nourished	*p*-Value
Gender	Female	28 (43.1)	18 (64.3)	10 (35.7)	0.890
	Male	37 (56.9)	22 (59.5)	15 (40.5)	
Marital status	Married	56 (86.2)	32 (57.1)	24 (42.9)	0.122
	Divorced/Widowed	8 (12.3)	7 (87.5)	1 (12.5)	
	Single	1 (1.5)	0 (0)	1 (100)	
Age (years)	18–39	3 (4.6)	3 (100)	0 (0)	0.079
	40–59	34 (52.3)	20 (58.8)	14 (41.2)	
	60+	28 (43.1)	16(47.8)	12(52.2)	
Governorate	Hawalli	17 (26.2)	7 (41.2)	10 (58.8)	0.146
	Farwaniya	14 (21.5)	8 (57.1)	6 (42.9)	
	Capital	14 (21.5)	11 (78.6)	3 (21.4)	
	Al-Ahmadi	11 (16.9)	8 (72.7)	3 (27.3)	
	Jahra	5 (7.7)	4 (80)	1 (20)	
	Mubarak Al-Kabeer	4 (6.2)	1 (25)	3 (75)	
Employment	Unemployed	15 (23.1)	11 (73.3)	4 (26.7)	0.415
	Employed	27 (41.5)	17 (63)	10 (37)	
	Retired	23 (35.4)	12 (52.2)	11 (47.8)	
Monthly income (KWD)	<300	6 (9.3)	3 (50)	3 (50)	0.437
	301–1000	24 (36.9)	17 (57.1)	7 (42.9)	
	>1000	35 (53.8)	20 (57.1)	15 (42.9)	
Nationality	Kuwaiti	32 (49.2)	19 (59.4)	13 (40.6)	1.000
	Non-Kuwaiti	33 (50.8)	20 (60.6)	13 (39.4)	
Level of education	High school or lower	37 (56.9)	25 (67.6)	12 (32.4)	0.281
	Diploma	8 (12.3)	3 (37.5)	5 (62.5)	
	Bachelor’s or higher	20 (30.8)	12 (60.0)	8 (40.0)	

Data are presented as number (percentage). Nutritional status was categorized using the full PG-SGA: well nourished (PG-SGA A) vs. malnourished (PG-SGA B or C). Comparisons were performed using the chi-square test. A *p*-value < 0.05 was considered statistically significant.

**Table 2:** Clinical characteristics of colorectal cancer patients—total sample, *n* = 65—according to nutritional status based on full PG-SGA.

		N (%)	Malnourished	Well Nourished	*p*-Value
Number of comorbidities	0	27 (42)	14 (51.9)	13 (48.1)	0.50
	1	19 (29)	12 (63.2)	7 (36.8)	
	≥2	19 (29)	13 (68.4)	6 (31.6)	
Current smoker	Yes	11 (16.9)	8 (72.7)	3 (27.3)	0.543
	No	54 (83.1)	31 (57.4)	23 (42.6)	
Stage of cancer	Early locally advanced	2 (3.1)	1 (50)	1 (50.0)	0.929
	Late locally advanced	11 (16.9)	7 (63.6)	4 (36.4)	
	Metastasized	52 (80.0)	31 (59.6)	21 (40.4)	
Treatment type	Chemotherapy	45 (69.2)	28 (62.2)	17 (37.8)	0.209
	Target therapy	4 (6.2)	4 (100)	0 (0)	
	Immunotherapy	1 (1.5)	1 (100)	0 (0)	
	Combination	15 (23.1)	7 (46.7)	8 (53.3)	
Stoma	Yes	23 (35.4)	18 (78.3%)	5 (21.7%)	0.050
	No	42 (64.6)	21 (50.0%)	21 (50.0%)	
BMI category	Underweight	2 (3.1)	2 (100)	0 (0)	0.0565
	Normal	25 (38.5)	19 (76)	6 (24)	
	Overweight	18 (27.7)	7 (38.9)	11 (61.1)	
	Obese	20 (30.8)	11 (55)	9 (45)	

Data are presented as number (percentage). Differences based on nutritional status were tested using the chi-square test. A *p*-value < 0.05 was considered statistically significant.

**Table 3:** Comparison of PG-SGA and PG-SGA SF component scores and nutrition impact symptoms by nutritional status.

	PG-SGA SF			PG-SGA	
Variable	Total Sample 65 (100)	At Risk 37 (56.9)	Not at Risk 28 (43.1)	Malnourished 39 (60) *	Well Nourished 26 (40)
**NIS**					
No appetite	33 (51)	27 (73)	6 (21)	27 (73)	6 (21)
Nausea	14 (22)	14 (38)	0 (0)	7 (17.9)	0 (0.0)
Dry mouth	20 (31)	19 (51)	1 (4)	19 (48.7)	1 (3.8)
**PG-SGA Total Score**	9.5 (6.0–15.0)	—	—	11.0 (8.5–13.0)	3.0 (0.25–4.75)
**PG-SGA SF Total Score**	7 (4–13)	11 (10–15)	3.5 (1–5)	—	—

Data are presented as median (interquartile range) for continuous variables or number (percentage) for categorical variables. Nutrition impact symptoms are reported as frequency (percentage). Nutritional status was classified using the full Patient-Generated Subjective Global Assessment (PG-SGA) and the PG-SGA Short Form (SF). PG-SGA A indicates well nourished; PG-SGA B/C indicates malnourished. A PG-SGA SF score ≥ 9 was used to define nutritional risk. Group comparisons were conducted using the Mann–Whitney U test and chi-square test. Statistical significance was set at *p* < 0.05. * Among malnourished patients, 16 (24.6%) were moderately malnourished (PG-SGA B), and 23 (35.4%) were severely malnourished (PG-SGA C).

**Table 4:** MST: Nutritional risk identification and component score analysis.

MST	Total Sample 65 (100)	At Risk 42 (64.6)	Not at Risk 23 (35.4)
Weight loss			
No	24 (37)	7 (19)	17 (61)
Unsure	14 (22)	11 (30)	3 (11)
1–5 kg	9 (14)	6 (16)	3 (11)
6–10 kg	6 (9)	5 (14)	1 (4)
11–15 kg	3 (5)	2 (5)	1 (4)
>15 kg	8 (12)	6 (16)	3 (11)
Poor appetite	42 (65)	31 (84)	11 (39)
**MST total score**	2 (0–3)	3 (2–3)	0 (0–2.25)

Data are presented as number (percentage) or median (interquartile range). Nutritional risk was defined as MST score ≥ 2. Group comparisons were performed using the chi-square test. A *p*-value < 0.05 was considered statistically significant.

**Table 5:** Comparison of anthropometric measures and weight history by nutritional status (PG-SGA classification).

Variable	Malnourished Median (IQR)	Well Nourished Median (IQR)	*p*-Value	Effect Size r
% weight loss in 1 month	1.25 (0.00–2.87)	0.00 (0.00–0.00)	0.001	0.42
% weight loss in 6 months	3.33 (0.00–13.77)	0.00 (0.00–1.78)	0.003	0.39
Mid-arm	27.35 (24.60–31.02)	31.20 (28.06–35.01)	0.01	0.31
Mid-calf	34.40 (32.10–37.62)	37.72 (34.74–40.05)	0.04	0.26
CBW (kg)	66.30 (58.00–81.30)	78.40 (67.95–91.05)	0.049	0.24
Weight (last month)	71.00 (57.50–82.00)	77.00 (65.00–87.50)	0.11	0.20
BMI (kg/m^2^)	24.50 (21.55–31.65)	28.70 (24.35–32.60)	0.15	0.18
Weight (3 months)	70.00 (57.00–81.00)	76.00 (67.25–85.25)	0.22	0.15
Weight (6 months)	74.00 (60.50–82.00)	74.00 (67.25–84.75)	0.55	0.07

Data are presented as median (interquartile range). Group differences were analyzed using the Mann–Whitney U test. A *p*-value < 0.05 was considered statistically significant. Effect sizes (r) were interpreted as small (0.10–0.29), medium (0.30–0.49), and large (≥0.50).

**Table 6:** Comparison of nutrient intake between malnourished and well-nourished patients (full PG-SGA).

Nutrient Intake	Malnourished Median (IQR)	Well Nourished Median (IQR)	*p*-Value
Energy intake (kcal)	1384.00 (1067.00–1762.50)	1731.00 (1287.50–2296.75)	0.03
Protein (g)	60.00 (43.00–78.50)	101.50 (76.25–128.00)	0.0001
Protein intake (g/kg)	0.82 (0.62–1.14)	1.27 (0.96–1.57)	0.004
Met EER, n (%)	16 (41.0)	12 (46.2)	0.88

Data are presented as median (interquartile range, IQR) for continuous variables and n (%) for categorical variables. The Mann–Whitney U test was used to compare nutrient intake variables between malnourished (PG-SGA B/C) and well-nourished (PG-SGA A) patients, while the chi-square test was used to compare the proportion of patients meeting estimated energy requirements. Estimated energy requirement (EER); gram per kilogram (g/kg); kilocalorie (kcal). Statistical significance was set at *p* < 0.05.

**Table 7:** Association between dietitian referral and nutritional status by full PG-SGA.

Seen by a Dietitian	Malnourished, *n* (%)	Well Nourished, *n* (%)	*p*-Value
Yes	19 (70.4)	8 (29.6)	0.24
No	20 (52.6)	18 (47.4)	

Data are presented as n (%). Nutritional status was categorized according to full PG-SGA interpretation, and the *p*-value was calculated using the chi-square test.

**Table 8:** NISs according to malnutrition status based on full PG-SGA.

Nutrition Impact Symptom	Malnourished *n* = 39	Well Nourished *n* = 26	*p*-Value
No appetite	27 (69.2)	6 (23.1)	0.001
Dry mouth	19 (48.7)	1 (3.8)	0.0004
Diarrhea	16 (41.0)	3 (11.5)	0.02
Nausea	14 (35.9)	0 (0.0)	0.002
Early Satiety	13 (33.3)	1 (3.8)	0.01
Constipation	13 (33.3)	5 (19.2)	0.34
Fatigue	11 (28.2)	2 (7.7)	0.09
Taste changes	11 (28.2)	2 (7.7)	0.09
Mouth sores	9 (23.1)	0 (0.0)	0.02
Pain	7 (17.9)	0 (0.0)	0.06
Vomiting	7 (17.9)	0 (0.0)	0.06
Problems swallowing	5 (12.8)	0 (0.0)	0.15
Hyperosmia	5 (12.8)	0 (0.0)	0.15

Data are presented as number (percentage). Group differences were analyzed using the chi-square test. Bonferroni correction was applied for multiple comparisons across 13 symptoms; *p*-values < 0.00385 were considered statistically significant.

**Table 9:** Multivariate logistic regression for predicting malnutrition (PG-SGA B/C vs. A).

Variable	Odds Ratio (OR)	95% Confidence Interval	*p*-Value
Dry mouth (yes vs. no)	17.65	2.02–154.19	0.009
Mid-arm muscle circumference (low vs. normal)	5.21	1.25–21.78	0.023
BMI (continuous)	0.94	0.84–1.04	0.19
Presence of stoma (yes vs. no)	1.78	0.51–6.12	0.34
Cancer stage (metastatic vs. early)	3.1	0.85–11.30	0.08
Age (per year)	1.01	0.97–1.06	0.64
Sex (female vs. male)	0.88	0.30–2.55	0.79

The model included statistically significant NISs (after Bonferroni correction) and key covariates (stoma status, BMI, cancer stage, sex, and age). Symptoms like vomiting and fatigue were excluded due to perfect separation (i.e., present only among malnourished patients). Model performance: AUC = 0.842, Nagelkerke R^2^ = 0.398, and AIC = 61.2.

**Table 10:** Diagnostic accuracy of MST and PG-SGA SF against full PG-SGA.

Metric	PG-SGA SF vs. PG-SGA	MST vs. PG-SGA
True Positives (TFs)	34.0	31.0
False Positives (FPs)	3.0	11.0
True Negatives (TNs)	23.0	15.0
False Negatives (FNs)	5.0	8.0
Sensitivity *	87.2% [73.3–94.4]	79.5% [64.5–89.2]
Specificity *	88.5% [71.0–96.0]	57.7% [38.9–74.5]
PPV *	91.9% [78.7– 97.2]	57.7% [38.9–74.5]
NPV *	82.1% [64.4–92.1]	65.2% [44.9–81.2]
Cohen’s Kappa (κ) *	0.75 [0.58–0.91]	0.38 [0.14–0.61]

* Estimate [95% CI]. Note: Sensitivity, specificity, positive predictive value (PPV), negative predictive value (NPV), and overall accuracy were calculated for the Malnutrition Screening Tool (MST) and the PG-SGA Short Form (SF), using the full PG-SGA classification as the reference standard (PG-SGA B/C vs. A). Statistical significance was set at *p* < 0.05.

## Data Availability

The data supporting the findings of this study are available from the corresponding author upon reasonable request. Data are not publicly available due to ethical and privacy restrictions.

## Supplementary Materials

The following supporting information can be downloaded at https://www.mdpi.com/article/10.3390/nu17172770/s1. Table S1. Comparison of PG-SGA SF and MST Domains and Features. Table S2. Comparison of PG-SGA and PG-SGA SF Component Scores and Nutrition Impact Symptoms by Nutritional Status. Table S3. Proportion of Patients Meeting Estimated Energy Requirements by Nutritional Status (Full PG-SGA Classification. Table S4. Comparison of Nutrient Intake Between Malnourished and Well-nourished Patients (Full PG-SGA). Table S5. Comparison of Biochemical Parameters Between Malnourished and Well-nourished Patients (Full PG-SGA)

## Abbreviations

The following abbreviations are used in this manuscript:

CRC	Colorectal Cancer
PG-SGA	Patient-Generated Subjective Global Assessment
PG-SGA SF	Patient-Generated Subjective Global Assessment Short Form
MST	Malnutrition Screening Tool
NIS	Nutrition Impact Symptoms
KCCC	Kuwait Cancer Control Center
BMI	Body Mass Index
NFPE	Nutrition-Focused Physical Examination
IBW	Ideal Body Weight
ABW	Adjusted Body Weight
RDA	Recommended Dietary Allowance
CI	Confidence Interval
OR	Odds Ratio
R	R Statistical Software
IQR	Interquartile Range
HIS	Hospital Information System
RBC	Red Blood Cells
EHR	Electronic Health Record
EER	Estimated Energy Requirements
MCH	Mean Corpuscular Hemoglobin
MCHC	Mean Corpuscular Hemoglobin Concentration
MCV	Mean Corpuscular Volume
RDW	Red Cell Distribution Width
PT-Global	Platform for PG-SGA scoring tool
