# Supplementary material for: Malnutrition and Nutrition Impact Symptoms in Kuwaiti Colorectal Cancer Patients: Validation of PG-SGA Short Form
Source: Nutrients. 2025 Aug 27;17(17):2770. doi: 10.3390/nu17172770 (PMC12430223; doi:10.3390/nu17172770)
Supplement: Supplementary file 1 [file nutrients-17-02770-s001.zip › nutrients-3805623-supplementary.pdf]

**Supplementary Material**

**Supplementary Table S1. Comparison of PG-SGA SF and MST Domains and Features**

| Domain / Item | PG-SGA SF | MST | Notes |
|---|---|---|---|
| Unintentional weight loss | ✓ | ✓ | Both assess this |
| Food intake reduction | ✓ | ✓ | MST asks if intake has decreased |
| Nutrition impact symptoms | ✓ | ✗ | Critical for cancer patients |
| Functional capacity | ✓ | ✗ | PG-SGA SF assesses impact on daily life |
| Symptoms duration/context | ✓ | ✗ | PG-SGA SF specifies time & impact |
| Patient self-assessment | ✓ | ✗ | PG-SGA SF empowers patient input |
| Total score-based categorization | ✓ | ✗ | PG-SGA SF stratifies severity |
| Requires clinician input | ✓ | ✗ | PG-SGA SF can be self-administered |
| Time to complete | ~5 min | <2 min | PG-SGA SF is longer but more informative |

**Note.** This table outlines the key differences and overlaps between the Patient-Generated Subjective Global Assessment Short Form (PG-SGA SF) and the Malnutrition Screening Tool (MST). While both tools assess unintentional weight loss and reduced food intake, only the PG-SGA SF incorporates nutrition impact symptoms, functional capacity, symptom duration, and patient self-assessment. Unlike MST, the PG-SGA SF provides a score-based severity classification and can be self-administered, though it takes slightly longer to complete.

**Supplementary Table S2.** Comparison of PG-SGA and PG-SGA SF Component Scores and Nutrition Impact Symptoms by Nutritional Status.

| | PG-SGA SF | | | PG-SGA | |
|---|---|---|---|---|---|
| **Variable** | **Total Sample 65(100)** | **At Risk 37(56.9)** | **Not At Risk 28(43.1)** | **Malnourished 39(60) *** | **Well-Nourished 26(40)** |
| **Box 1: Weight Score** | 0 (0–1) | 1 (0–1) | 0 (0–0) | 1.0 (0–1) | 0.0 (0–0) |
| **Box 2: Food Intake Score** | 0 (0–1) | 1 (0–1) | 0 (0–0) | 1.0 (0–1) | 1.0 (0–1) |
| **Box 3: NIS Score** | 4 (1–7) | 7 (6–9) | 1 (0–3) | 7.0 (5–9) | 1.0 (0–3) |
| No problem eating | 11 (17) | 1 (3) | 10 (36) | 2 (5.1) | 9 (34.6) |
| No appetite | 33 (51) | 27 (73) | 6 (21) | 27 (73) | 6 (21) |
| Nausea | 14 (22) | 14 (38) | 0 (0) | 7 (17.9) | 0 (0.0) |
| Constipation | 18 (28) | 12 (32) | 6 (21) | 13 (33.3) | 5 (19.2) |
| Mouth sores | 9 (14) | 9 (24) | 0 (0) | 9 (23.1) | 0 (0.0) |
| Dysgeusia/Aguseia | 13 (20) | 11 (30) | 2 (7) | 11 (28.2) | 2 (7.7) |
| Problems swallowing | 5 (8) | 5 (14) | 0 (0) | 5 (12.8) | 0 (0.0) |
| Pain | 7 (11) | 7 (19) | 0 (0) | 0 (0.0) | 7 (10.8) |
| Vomiting | 7 (11) | 7 (19) | 0 (0) | 7 (17.9) | 0 (0.0) |

| | | | | | |
|---|---|---|---|---|---|
| Diarrhea | 19 (29) | 17 (46) | 2 (7) | 16 (41.0) | 3 (11.5) |
| Dry mouth | 20 (31) | 19 (51) | 1 (4) | 19 (48.7) | 1 (3.8) |
| Hyperosmia | 5 (8) | 5 (14) | 0 (0) | 5 (12.8) | 0 (0.0) |
| Early satiety | 14 (22) | 13 (35) | 1 (4) | 13 (33.3) | 1 (3.8) |
| Fatigue | 13 (20) | 11 (30) | 2 (7) | 11 (28.2) | 2 (7.7) |
| Others | 17 (26) | 12 (32) | 5 (18) | 12 (30.8) | 5 (19.2) |
| **Box 4: Function Score** | 1 (0–3) | 3 (1–3) | 0.5 (0–1.25) | 3.0 (1.0–3) | 0.5 (0.0–1) |
| **Box 5: Disease-Nutritional Requirements Score** | 1 (1–2) | — | — | 1 (1–2) | 1 (1–1) |
| **Box 6: Metabolic Demand Score** | 0 (0–0) | — | — | 0 (0–0) | 0 (0–0) |
| **Box 7: Physical Exam Score** | 1 (1–2) | — | — | 2 (1–2) | 0 (0–1) |
| **PG-SGA Total Score** | 9.5 (6.0–15.0) | — | — | 11.0 (8.5–13.0) | 3.0 (0.25–4.75) |
| **PG-SGA SF Total Score** | 7 (4–13) | 11 (10–15) | 3.5 (1–5) | — | — |

Data are presented as median (interquartile range) for continuous variables or number (percentage) for categorical variables. Nutrition impact symptoms are reported as frequency (percentage). Nutritional status was classified using the full Patient-Generated Subjective Global Assessment (PG-SGA) and the PG-SGA Short Form (SF). PG-SGA A indicates well-nourished; PG-SGA B/C indicates malnourished. A PG-SGA SF score ≥9 was used to define nutritional risk. Group comparisons were conducted using the Mann–Whitney U test and chi-square test. Statistical significance was set at $p < 0.05$. *Among malnourished patients, 16 (24.6%) were moderately malnourished (PG-SGA B) and 23 (35.4%) were severely malnourished (PG-SGA C).

**Supplementary Table S3. Proportion of Patients Meeting Estimated Energy Requirements by Nutritional Status (Full PG-SGA Classification)**

| Nutritional Status | Met Energy Requirements, n (%) | Did Not Meet, n (%) | p-value |
|---|---|---|---|
| Well-nourished | 12 (46.2) | 14 (53.8) | 0.88 |
| Malnourished | 16 (41.0) | 23 (59.0) | |

Note: data are presented as N (%). Energy adequacy is defined based on estimated energy needs (e.g., 25–30 kcal/kg/day). P-value calculated using the Chi-square test. PG-SGA = Patient-Generated Subjective Global Assessment.

**Supplementary Table S4.** Comparison of Nutrient Intake Between Malnourished and Well-nourished Patients (Full PG-SGA).

| **Nutrient Intake** | **Malnourished Median (IQR)** | **Well-Nourished Median (IQR)** | **p-value** |
|---|---|---|---|
| **Energy Intake (kcal)** | 1384.00 (1067.00–1762.50) | 1731.00 (1287.50–2296.75) | **0.03** |
| **Protein %** | 18.42 (13.33–22.44) | 22.13 (17.52–29.34) | **0.04** |
| **Protein (g)** | 60.00 (43.00–78.50) | 101.50 (76.25–128.00) | **0.0001** |
| **Protein intake (g/kg)** | 0.82 (0.62–1.14) | 1.27 (0.96–1.57) | **0.004** |
| Carbohydrates % | 55.77 (47.93–61.61) | 51.81 (43.85–58.52) | 0.33 |
| Carbohydrates intake (g/day) | 167.00 (120.50–259.50) | 247.50 (153.75–311.00) | 0.10 |
| Fat % | 23.97 (18.86–32.18) | 23.48 (19.41–32.18) | 0.92 |

| | | | |
|---|---|---|---|
| Fat intake (g/day) | 37.10 (21.20–58.50) | 42.50 (34.80–66.25) | 0.24 |
| Met EER, n (%) | 16 (41.0) | 12 (46.2) | 0.88 |

Note. Data are presented as median (interquartile range, IQR) for continuous variables and n (%) for categorical variables. The Mann–Whitney U test was used to compare nutrient intake variables between malnourished (PG-SGA B/C) and well-nourished (PG-SGA A) patients, while the chi-square test was used to compare the proportion of patients meeting estimated energy requirements. Macronutrient intake is shown as both absolute intake (g/day) and percentage of total energy. Estimated Energy Requirement (EER); gram per kilogram (g/kg); kilocalorie (kcal). Statistical significance was set at $p < 0.05$.

**Supplementary Table S5: Comparison of biochemical parameters between malnourished and well-nourished patients (Full PG-SGA).**

| Biochemical Parameter | Malnourished<br>Median (IQR) | Well-Nourished<br>Median (IQR) | p-value |
|---|---|---|---|
| **Neutrophils(%)** | 63.90 (50.45–73.10) | 52.30 (48.80–57.60) | **0.004** |
| Lymphocytes(%) | 23.10 (14.90–38.35) | 31.30 (27.10–37.90) | 0.02 |
| Magnesium (mmol/L) | 0.76 (0.68–0.80) | 0.84 (0.73–0.87) | 0.02 |
| RBC (10*12/L) | 4.15 (3.65–4.75) | 4.62 (4.25–4.79) | 0.05 |
| Hct (L/L) | 0.36 (0.33–0.40) | 0.39 (0.35–0.42) | 0.06 |
| Hb(g/L) | 113.00 (103.00–128.50) | 129.00 (112.00–135.00) | 0.09 |
| Total Protein(g/L) | 68.00 (62.50–71.00) | 70.00 (67.00–72.00) | 0.11 |
| MPV (fL) | 9.80 (8.80–10.00) | 9.00 (7.80–9.70) | 0.11 |
| Basophils (%) | 0.50 (0.30–0.90) | 0.70 (0.50–0.80) | 0.13 |
| Glucose(mmol/L) | 5.59 (5.11–7.60) | 6.11 (5.41–8.32) | 0.17 |
| NRBC (#) | 0.00 (0.00–0.00) | 0.00 (0.00–0.00) | 0.19 |
| Potassium (mmol/L) | 4.20 (3.90–4.55) | 4.30 (4.10–4.70) | 0.21 |
| Albumin (g/L) | 37.00 (32.00–40.50) | 39.00 (36.00–41.00) | 0.25 |
| WBC (10*9/L) | 7.13 (5.35–8.90) | 5.75 (5.20–7.35) | 0.26 |
| Chloride (mmol/L) | 106.00 (104.50–108.00) | 108.00 (105.00–109.00) | 0.35 |
| Calcium (mmol/L) | 2.29 (2.17–2.37) | 2.31 (2.24–2.39) | 0.38 |
| Monocytes(%) | 9.40 (7.65–10.90) | 9.40 (8.00–12.00) | 0.41 |
| Urea (mmol/L) | 4.30 (3.65–6.05) | 3.80 (3.40–5.20) | 0.45 |
| ALP (U/L) | 143.00 (82.00–183.50) | 126.00 (91.00–171.00) | 0.47 |
| Eosinophils (%) | 2.20 (1.00–3.95) | 2.50 (1.80–3.60) | 0.55 |
| GGT (IU/L) | 47.00 (28.50–109.50) | 43.50 (22.25–89.75) | 0.58 |
| MCH (pg) | 28.30 (24.80–30.10) | 27.80 (25.10–29.30) | 0.59 |
| Sodium (mmol/L) | 140.00 (138.00–141.00) | 139.00 (138.00–141.00) | 0.61 |
| RDW (%) | 16.70 (15.05–19.65) | 16.70 (15.40–20.80) | 0.62 |
| Total Bilirubin (umol/L) | 8.20 (5.20–13.70) | 8.00 (5.70–10.20) | 0.62 |
| AST (U/L) | 26.00 (21.50–39.00) | 28.00 (20.00–31.00) | 0.64 |
| MCHC (g/L) | 320.00 (310.50–328.50) | 321.00 (310.00–331.00) | 0.64 |
| ALT (U/L) | 20.00 (14.00–35.50) | 20.00 (16.00–30.00) | 0.66 |
| Adjusted Calcium (mmol/L) | 2.41 (2.37–2.48) | 2.42 (2.35–2.44) | 0.69 |
| MCV (fL) | 87.40 (79.95–93.20) | 85.60 (80.00–91.40) | 0.77 |
| Phosphorus (mmol/L) | 1.12 (0.97–1.21) | 1.10 (0.96–1.21) | 0.81 |
| eGFR(mL/min/1.73 m2) | 99.00 (77.50–104.00) | 99.00 (87.00–102.00) | 0.84 |
| Platelets(10*9/L) | 223.50 (166.00–311.75) | 216.00 (194.00–289.00) | 0.92 |
| Creatinine (umol/L) | 66.00 (54.00–78.00) | 64.00 (58.00–79.00) | 0.99 |

Data are presented as median (interquartile range, IQR).The Mann–Whitney U test was used to compare biochemical parameters between malnourished (PG-SGA B/C) and well-nourished (PG-SGA A) patients. Statistical significance was set at $p < 0.05$. Red Blood Cells (RBC); Hematocrit (Hct); Hemoglobin (Hb), Mean Platelet Volume(MPV); Nucleated Red Blood Cells (NRBC); White Blood Cells (WBC); Alkaline Phosphatase (ALP); Gamma-Glutamyl Transferase (GGT); Mean Corpuscular Hemoglobin (MCH); Red Cell Distribution Width (RDW); Aspartate Aminotransferase (AST), Mean Corpuscular Hemoglobin Concentration

(MCHC); Alanine Aminotransferase (ALT); Mean Corpuscular Volume (MCV); Estimated Glomerular Filtration Rate (eGFR).

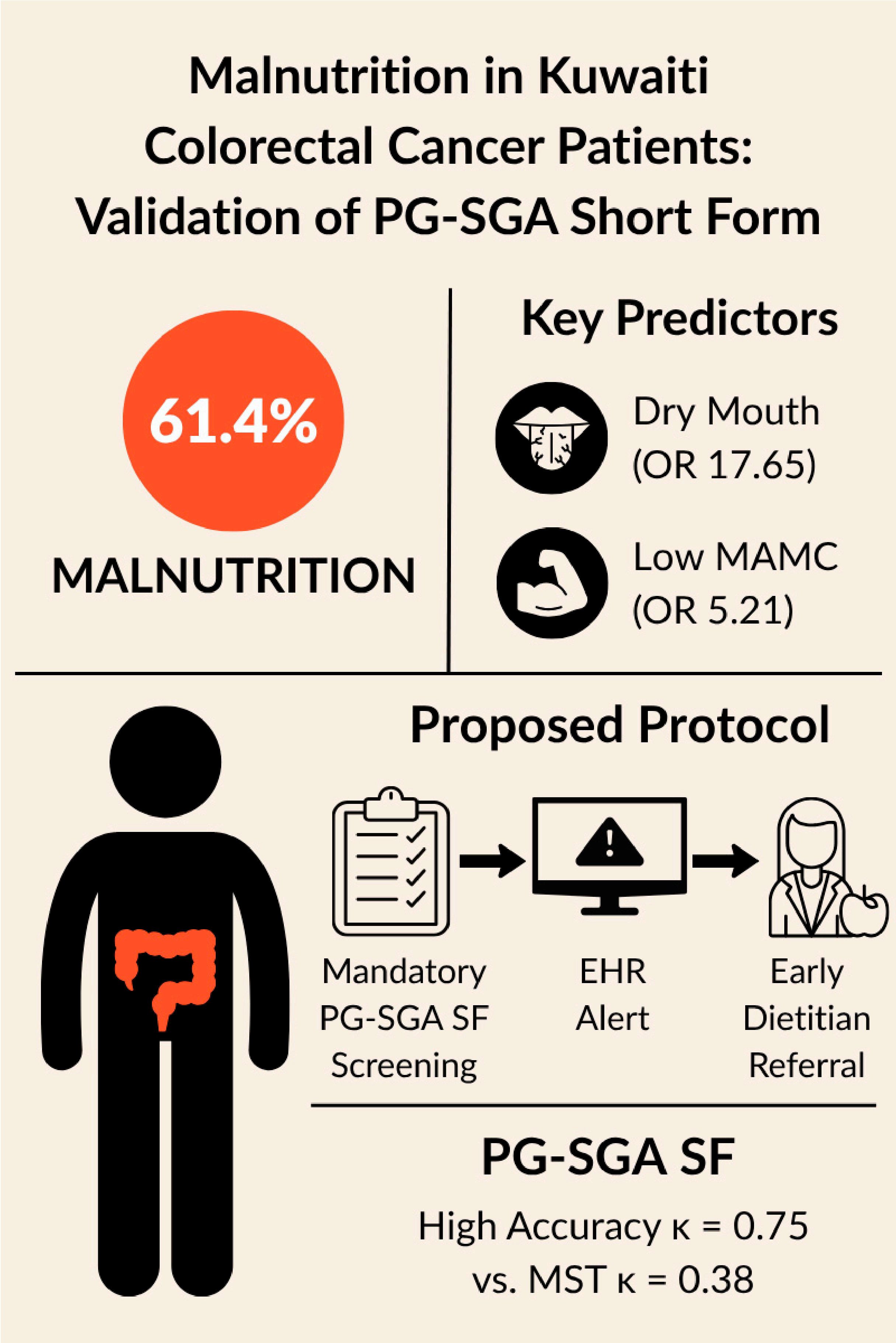

Malnutrition in Kuwaiti Colorectal Cancer Patients: Validation of PG-SGA Short Form
61.4%
MALNUTRITION
Key Predictors
Dry Mouth (OR 17.65)
Low MAMC (OR 5.21)
Proposed Protocol
Mandatory PG-SGA SF Screening
EHR Alert
Early Dietitian Referral
PG-SGA SF
High Accuracy κ = 0.75
vs. MST κ = 0.38
